# Study on *Salmonella* Isolates from Fresh Milk of Dairy Cows in Selected Districts of Wolaita Zone, Southern Ethiopia

**DOI:** 10.1155/2023/6837797

**Published:** 2023-02-23

**Authors:** Isayas Asefa, Ermias Legabo, Tsegaye Wolde, Haben Fesseha

**Affiliations:** School of Veterinary Medicine, Wolaita Sodo University, P.O. Box 138, Wolaita Sodo, Ethiopia

## Abstract

*Salmonella* infections are most commonly found in animal-derived foods. From December 2021 to May 2022, the researchers conducted a cross-sectional study to determine the prevalence of *Salmonella* isolated from raw milk collected in and around Areka town, Boloso Sore Woreda, Wolaita Zone, Southern Ethiopia. A total of 151 direct udder milk samples were collected at random and examined using bacteriological methods. The overall prevalence of *Salmonella* was 9.3% (14/151). Breed, age, body condition, lactation stage, and parity were statistically significant risk factors (*p* < 0.05). Salmonellosis was more common and statistically significant in dairy cows with poor body condition and late lactation stage, as well as the Holstein Friesian crossbreed, accounting for 17.6%, 19.1%, and 17.3%, respectively. The farm's husbandry hygiene and management system, on the other hand, had no significant association with salmonellosis (*p* > 0.05). Salmonellosis was generally considered to be moderately prevalent and was one of the diseases of dairy cows in the study area that could have an impact on dairy production and have serious health and financial repercussions. As a result, improvements in milk quality maintenance and assurance are encouraged, and the need for additional research in the study area was suggested along with other ideas.

## 1. Introduction

*Salmonella* is a Gram-negative, facultatively anaerobic, nonsporulating straight rod genus in the Enterobacteriaceae family [1, 2]. They are intracellular facultative pathogens that range in size from 2 to 3 m. The rod's shape is maintained by the bacterial cytoskeleton, which is made up of an actin-like protein [3]. This organism has the potential to be pathogenic to both humans and animals. *Salmonella enterica* and *Salmonella bongori* are two bacteria that cause infectious diseases in humans and animals, with one or more of three major syndromes: septicemia, acute enteritis, and chronic enteritis [4].

Salmonellosis is common in cattle. They are frequently concerned because of cattle disease and the possibility of infecting humans who come into contact with cattle or consume dairy or bovine meat products. As a result, dairy cattle infected with nontyphoidal *Salmonella* spp. can pose a significant risk to public health [5]. It is a common and important foodborne pathogen that causes salmonellosis (enteric fever, paratyphoid) in humans and animals, with serious medical and economic consequences. Salmonella infections are most commonly found in food animals such as pigs, poultry, and cattle. *Salmonella* contamination of animals and animal products on farms, as well as organs and carcasses in abattoirs, is a major cause of the pathogen's spread [6].

Although the majority of human infections cause mild gastroenteritis, life-threatening systemic infections are common, particularly among the elderly. Invasive nontyphoid *Salmonella* infects newborns, children, the elderly, and immunocompromised adults all over the world, but particularly in Africa, where co-infection with malaria or the human immunodeficiency virus (HIV) worsens the illness [7, 8].

Salmonellosis, the clinical form of *Salmonella* infection, is a costly disease for dairy producers because of deaths, treatment costs, decreased milk yield, and herd weight loss. Cattle infected with *Salmonella* may be clinically or subclinically ill, causing the bacteria to be released in their faces. As a result, dairy farmers must be aware that *Salmonella* can be found in seemingly healthy cows on their farms, which is critical in terms of food safety concerns [9]. Infected animals and human feces are significant sources of bacterial contamination in the environment and food [10].

A higher incidence of *Salmonella* shedding in dairies has been linked to several factors. Weather, population density, land use, farming exercise, meal harvesting and processing technology, and consumer habits all influence disease incidence and prevalence [11]. Typhoid fever kills 16 million people each year, gastroenteritis kills 1.3 billion people, and *Salmonella* kills 3 million people [12].

A decade ago, there was an alarming increase in the prevalence of antibiotic-resistant*Salmonella*, which could be attributed to the selective pressure associated with the use of antimicrobial agents in animal feed [13]. Foods derived from animals are a source of multidrug-resistant*Salmonella serovars* [14]. Antimicrobial usage in animal feeds is frequently at subtherapeutic or prophylactic levels, which may promote the on-farm selection of antibiotic-resistant strains and significantly increase the risk of infection associated with the consumption of contaminated meat and milk products [15, 16].

The primary goal of *Salmonella* treatment should be to correct dehydration caused by prolonged diarrhea by replacing fluids and electrolytes [17]. Proper food preparation reduces the risk of infection, and to avoid *Salmonella* infection in humans, hands should be thoroughly washed with soap and water after handling meat [18].

Several studies conducted in Ethiopia revealed a high prevalence of *Salmonella* and antibiotic resistance patterns in both veterinary and public settings [16, 19, 20]. However, the impact of those investigations on the national perspective, particularly on raw cow milk, is much more limited in this study area. Thus, determining the prevalence and risk factors of *Salmonella* from fresh raw milk of dairy cows in the Boloso Sore district was the goal of this research.

## 2. Methods and Materials

### 2.1. Study Area

**Figure 1:**
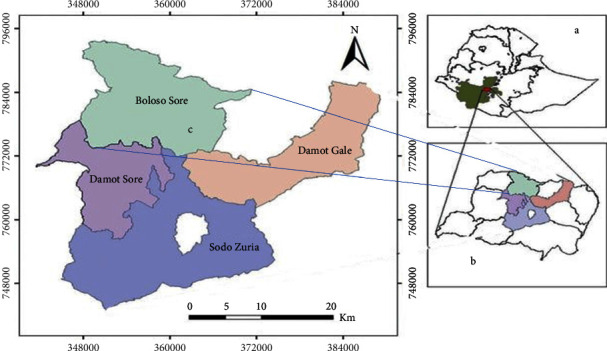
Map of the study area Ethiopia (a), Wolaita Zone (b), and Boloso Sore district (c).

Boloso Sore district is located 380 kilometers southwest of Ethiopia's capital city, Addis Ababa, in the Wolaita Zone of the Southern Nations, Nationalities, and Peoples' Region. It is bounded on the west by Boloso Bombe, on the north by Kembata Tembaro, on the south by Sodo Zuria and Damot Sore, and on the east by Damota Gale. Based on the climate, 80% of the population lived in Woina Dega (mid-altitude), the rest in Kola (lowland), and a negligible percentage in Dega (Highland). The site is located at 7°05′ N 37°40′ E/7.083° N 37.667° E and is 1350–2380 m above sea level (Figure 1). With an average annual rainfall of 1300 mm and an average daily temperature of 20.4°C, a short-wet season lasts from March to May, and a long rainy season lasts from June to September. According to a 2016 report from the Wolaita Zone Livestock and Fishery Resources office, the livestock population of the Boloso Sore district was estimated to be 84,391 cattle, 57,331 ovines, 8396 caprines, 7321 equines, and 91,375 poultry [21, 22].

### 2.2. Study Population

The animals used in this study were healthy dairy cattle from smallholder dairy farms in the study area. The farms were chosen at random from data obtained from the Boloso Sore Woreda Agriculture and livestock resource development office on the number of dairy farms in the area. The dairy cows were classified according to their geographical locations (4 Kebeles), and the dairy farms (3 farms in each kebele) within them were managed semi-intensively, intensively, and extensively, and local Zebu and Holstein Friesian (HF) crossbreds were reared. Kebele and farms were chosen at random. In this study, risk factors included the management system, parity, lactation stage, husbandry hygiene, breed, age, and body condition of animals. The cows' ages were determined by observing their dentition characteristics and were classified as 3–6 years, 7–9 years, and >9 years [23].

Parity was divided into three categories: 1-2 calves (few), 3–6 calves (moderate), and more than 6 calves (many) [24]. Feeding, watering, and husbandry hygiene practices were classified as good (if there is a practice of feeding and watering animals in individual feed and water troughs and washing and drying udder with separate towels, milking healthy and young cows first) or poor (if there is a practice of collective feeding and watering of animals and washing and drying udder with separate towels, and milking with order is not practiced) [24]. The cow's lactation stage was also divided into three categories: early-stage lactation (1–4 months), mid-stage lactation (>4–8 months), and late-stage lactation (above 8 months) [23].

### 2.3. Study Design

A cross-sectional study was carried out in and around Areka town, Boloso Sore Woreda, from December to May 2022, to isolate *Salmonella* from dairy herd milk samples and identify related risk factors.

### 2.4. Sample Size Determination

The prevalence of 10.76% from prior studies [25] was used to calculate the sample size using the Thrusfield [26] formula at a 5% level of significance and a 95% confidence interval.

(1) $$\begin{array}{c} \frac{1.96^{2}\times Pexp\left(1-Pexp\right)}{d^{2}}, \end{array}$$

where *n* = required sample size, Pexp = expected prevalence, and *d* = desired absolute precision.

Based on the above formula, the minimum sample size was 148 dairy cows. However, 151 different milk samples were used.

### 2.5. Sample Collection and Transportation

After vigorously scrubbing the teats with a pledge of cotton moistened (but not completely wet) with 70% ethyl alcohol and drying up, a direct udder milk sample was collected from apparently healthy lactating cows. The first 3-4 streams of milk were discarded, and 15 to 20 mL of milk was aseptically collected from all quarters of each cow in a sterile screw-cupped bottle. The samples were properly coded and immediately transported under cold conditions via an ice box to the Wolaita Sodo University School of Veterinary Medicine's Microbiology Laboratory for cultural examination. When the samples arrived, they were immediately cultured or stored at +4°C for a maximum of 24 hours before being examined. During face-to-face communication with farm managers or representative individuals, a data recording sheet was used to collect information on the cow's age, parity, breed, lactation stage, milking, feeding and watering hygiene, farm cleanliness, and body condition.

### 2.6. Isolation and Identification of *Salmonella*

The techniques used to isolate and identify *Salmonella* were recommended by the International Organization for Standardization (ISO-6579, 2002) and the World Health Organization [27]. Global foodborne infections network (formerly WHO global *Salmonella* Surveillance) [27]. In a nonselective liquid medium (buffered peptone water (BPW) (Oxoid CM509, Basingstoke, England), a 10 ml milk sample was mixed with 90 ml of pre-enrichment, and the sample mixture was thoroughly shaken before being incubated at 37°C for 24 hours. Following incubation, the culture was mixed, and a portion (0.1 ml) was transferred to a tube containing 10 ml of selective enrichment liquid medium (Rappaport Vassiliadis (RV)) broth and incubated at 41.5°C for 24 hours. A 10 *µ*l of loop full inoculum from selective enrichment media was streaked onto Xylose Lysine Deoxycholate (XLD) (Oxoid CM0469, Basingstoke, England) agar and *Salmonella Shigella* (SS) agar plates prepared on petri-dishes and incubated at 37 ± 1°C for 24 ± 3 hours. After proper incubation, the plates were examined for the presence of typical *Salmonella* colonies. The typical colonies of *Salmonella* grown on Xylose Lysine Deoxycholate agar medium produce black centers with distinct red colonies due to the color change of phenol red in medium and colorless transparent colonies on *Salmonella Shigella* agar. The presumptive *Salmonella* colonies on the XLD (Oxoid CM0469, Basingstoke, England) and SS agar medium were transferred onto the surface of predried nutrient agar plates in a manner that allow isolated colonies to develop and incubated at 37°C for 24 hours in further confirmation with biochemical tests. Thus, all suspected *Salmonella* colonies were picked from the nutrient agar and inoculated into the biochemical test including Triple Sugar Iron (TSI) agar (Oxoid CM0277, Basingstoke, England) for the TSI test, Simmons's Citrate agar (Oxoid CM53, Basingstoke, England) for the citrate utilization test, Tryptone Soya Broth (Becton Dickinson, USA) for the indole test and Methyl red-Voges Proskauer (MR-VP) (Micromaster Thane, India) for methyl red and Voges Proskauer test and incubated for 24 or 48 hours at 37°C. Colonies producing an alkaline (red) slant, with acid (yellow) but on TSI with blackening or hydrogen sulfide production, negative for Tryptophan utilization on indole test (yellow-brown ring), positive for Methyl red (produce red color on the surface of medium), negative for Voges–Proskauer (yellow color), and positive for Citrate utilization (deep blue slant) were consider to be *Salmonella* positive [28, 29].

### 2.7. Data Analysis

Microsoft Office Excel 2010 was used to enter and save the data, and STATA 14 for Windows was used to conduct the analysis (Stata Corporation, Texas, USA, 2014). Descriptive statics such as percentage was used to determine the level of prevalence. The associations between the various potential risk factors of animals assed by the Pearson chi-square (*χ*^2^) and logistic regressions were used. A *p* < 0.05 was considered significant.

### 2.8. Ethical Considerations

Ethical consent was obtained from Wolaita Sodo University, Research Review Committee (with the reference number WSU 41/22/2342/2022) to collect research and conduct the research and the committee approved this research work. The purpose of the study was explained to the local leaders and farm owners before taking the samples, and informed consent was obtained to take the appropriate sample through verbal consent.

## 3. Results

### 3.1. The Overall Prevalence of *Salmonella* in Dairy Cows

**Figure 2:**
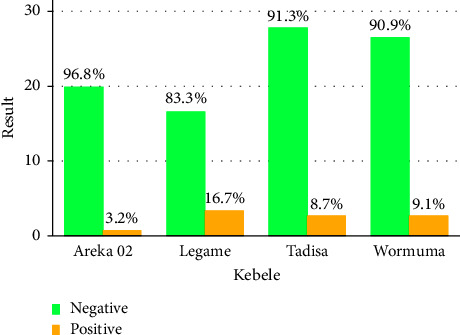
The overall prevalence of *Salmonella* in the study area.

A total of 151 milk samples were tested for *Salmonella* using the bacteriological method. *Salmonella* was found in 14 of these samples, with a 9.3% overall prevalence. Similarly, the prevalence of this study area's Kebeles, Areka 02, Legama, Tadisa, and Wormuma were 3.2%, 16.7%, 8.7%, and 9.1%, respectively (Figure 2).

*Salmonella* prevalence in the study area was found to be significantly associated with the lactation stage, parity, breed, and body condition of animals (*p* < 0.05). In the current study area, however, no statistically significant difference was found between husbandry hygiene and farm management systems (Table 1).

## 4. Discussion

The current study examined 151 milk samples, 14 of which tested positive for *Salmonella*, yielding an overall prevalence of 9.3%. This finding agreed with the prevalence reports of 10.42% [30], 12.9% [24], 10.76% [20], 8% [31], and 12.5% [31, 32]. This agreement could be attributed to the agroecological similarity of the study area and the similarity of the protocol of bacterial isolation and identification techniques.

The current finding, however, was higher than the 3.7% found by Ketema et al. [33], the 6.50% found by Beyene et al. [34]; and the 4.95% found by Amera et al. [35]. The current study figures, on the other hand, were significantly lower than the findings of Azage and Kibret [36], who reported 70% from cattle meat samples in Bahir Dar, and the same prevalence rate was also reported from dairy cattle fecal samples in central Texas, USA [37]. These *Salmonella* isolation rate differences could be attributed to differences in sample type, housing conditions; cattle feed types, and dairy cow feeding and watering habits between dairy farms and countries.

The current study found that the age of the animals was statistically significant and that there were strong relationships between age categories and the rate of *Salmonella* detection. Furthermore, the study found that the rate of *Salmonella* isolation was higher in animals over the age of 9 years than in other age groups. This finding was supported by [38], who discovered a strong association between age and the prevalence of bacteria. A significant difference in the distribution of *Salmonella* isolates was observed between body condition categories (*X*^2^ = 7.953 and *p*=0.019).

According to the current study's findings, animals with poor body conditions had more *Salmonella* in their milk than animals with good and medium body conditions. This could be because the body condition of animals is reduced when they are infected with parasites, feed, and water deprivation, and stress create favorable conditions for pathogens to multiply in the body of animals [39, 40]. This could also be associated with an animal's immune system's weakened ability to defend against infection-causing agents as they age [41].

In the current study, we also attempted to compare the rate of *Salmonella* isolation among farms with the varying breeds, lactation stages, and parity. Accordingly, the prevalence of *Salmonella* isolates was high in HF cross (17.3%) than in local Zebu (5%); highest in the late lactation stage (19.1%) and followed by mid (5.9%) and early (2.8%) lactation stage; as well as highest in those animals with many parities (44.4%) and followed by moderate (7.3%), and few (6.7%) parities. This could be partly due to the types of animal breeding (physiological difference), lactation stage (as lactation stage increased so did *Salmonella* prevalence), and parities. Also, milk handling, and management practices and the likelihood of contamination are considered high in the late lactation stage and many parity animals since *Salmonella* are ubiquitous [42, 11].

Moreover, in this study, there was a statistically significant association between *Salmonella* occurrence and lactation stage and parity (*X*^2^ = 14.09 and *p*=0.001). A cow with many categories of parity 4 had the highest *Salmonella* isolation rate (44.4%). When compared to animals with a moderate level of parity, those with fewer categories of parity have a more effective defense mechanism [42]. The reason could be the increased chance of infection time and the prolonged duration of infection on the animal body, particularly in a herd lacking appropriate disease prevention and control programs [11].

It is well known that raw milk for public consumption and the consumption of raw milk-based products pose some risks if not properly pasteurized. Contamination of raw milk can occur from the environment, particularly during milking and milk handling, as well as from water and milking equipment and facilities [43]. *Salmonella* occurrences appeared to be high in the milk samples tested here, and the possibility of this organism growing in improperly handled milk and products made from raw milk poses a public health risk, particularly to vulnerable members of the population.

### 4.1. Limitations of the Study

The current study used conventional cultural and biochemical identification methods to isolate *Salmonella* from dairy cow's milk and recommended further research to detect and identify *Salmonella* by the PCR-based method. Continuing milk surveys will aid in estimating the true level of risk associated with these practices and may aid in the identification of dairy farm management practices that reduce milk contamination with zoonotic foodborne pathogens. Therefore, for the detection of *Salmonella* in milk with better accuracy and specificity within a short period polymerase chain reaction method is recommended.

## 5. Conclusion

To summarize, *Salmonella* is a well-known and widespread foodborne pathogen that causes salmonellosis (enteric fever, paratyphoid) in both animals and humans, with serious health and economic consequences. The current study found a moderate prevalence of *Salmonella* in animals at smallholder dairy farms in the study area. The findings, therefore, showed that cow milk that has been taken directly from the udder can contain *Salmonella*. Additionally, it foresaw the various risk factors that increase the animal's vulnerability to *Salmonella* infection. Similarly, dairy cows exposed to these risk factors were more susceptible to Salmonellosis. In general, the findings of this study provide insights into the magnitude of potential health risks related to the consumption of raw milk. Thus, further research on determining antimicrobial resistance and serotyping of *Salmonella* isolates, detailed epidemiological studies and periodic surveillance of *Salmonella* carrier animals, and training, education, and preparation guidelines to ensure the quality of raw milk for public consumption are required. The study area's dairy farmers and raw milk vendors should also take serious precautions to prevent *Salmonella* contamination of the milk. Additionally, it is important to support the active efforts of the veterinary departments in each district to educate dairy farmers on proper milk-handling techniques. To pinpoint the *Salmonella* serotypes that are prevalent in the study area, additional molecular research is also required.

## Figures and Tables

**Table 1:** Risk factors associated with the prevalence of *Salmonella* identified from milk samples in the study area.

Factors	Frequency	Prevalence	*χ* ^2^	*p* values
Breed			6.0890	0.014
HF cross	52	9 (17.3%)		
Local Zebu	99	5 (5%)		
Age			14.1208	0.001
3–6 years	62	4 (6.5%)		
7–9 years	80	6 (7.5%)		
>9 years	9	4 (44.4%)		
Body condition			7.9531	0.019
Good	34	1 (2.9%)		
Medium	66	4 (6.1%)		
Poor	51	9 (17.6%)		
Management			0.6704	0.715
Intensive	1	0 (0%)		
Semi-intensive	22	3 (13.6%)		
Extensive	128	11 (8.6%)		
Husbandry hygiene			1.7019	0.192
Good	15	0 (0%)		
Poor	136	14 (10.3%)		
Lactation stage			8.1844	0.017
Early	36	1 (2.8)		
Mid	68	4 (5.9%)		
Late	47	9 (19.1%)		
Parity			14.0926	0.001
Few	60	4 (6.7%)		
Moderate	82	6 (7.3%)		
Many	9	4 (44.4%)		

## Data Availability

All the datasets generated or analyzed during this study are included in this manuscript.

## Additional Points

Samples collection was performed by a veterinarian concerning and preservation of animal health and welfare.
